# Analysis of Network Structures in Thiol-Ene UV Curing System Using Reworkable Resins

**DOI:** 10.3390/polym11010005

**Published:** 2018-12-20

**Authors:** Haruyuki Okamura, Masashi Yamagaki, Kyohei Nakata

**Affiliations:** Department of Applied Chemistry, Graduate School of Engineering, Osaka Prefecture University, 1-1, Gakuen-cho, Naka-ku, Sakai, Osaka 599-8531, Japan; su108070@edu.osakafu-u.ac.jp (M.Y.); sv108038@edu.osakafu-u.ac.jp (K.N.)

**Keywords:** crosslinking, degradation, reworkability, UV curing, thiol-ene reaction, chain length

## Abstract

An analysis of the network structures in thiol-ene UV curing resins was carried out using reworkable resins composed of di(meth)acrylate monomers having tertiary ester linkages. The effect of the functionality of the thiols, the functional ratio of the thiol and ene functions, their conversions and curing atmosphere on the chain lengths was discussed. A mixture of (meth)acrylates, thiol compounds, a photoradical initiator, and a photoacid generator was cured by irradiation at 365 nm. The cured samples were degraded by heating after irradiation at 254 nm. Size exclusion chromatography (SEC) and ^1^H NMR analyses of the degraded samples were carried out after the methylation. The crosslinking conditions strongly affected the network structures. The degraded samples have molecular weights between 250 and 2700. The molecular weights of the degraded resins increased with the functionality of the thiol compounds. The chain length dependence suggests that thiol compounds with a high functionality have a low reactivity due to steric hindrance. The chain lengths of the degraded networks were nearly proportional to the concentration of the (meth)acrylate monomers. The addition of reactive diluents enhanced the reactivity and increased the chain length.

## 1. Introduction

Degradable thermosets [1,2,3,4,5,6,7,8,9,10,11,12,13,14] have attracted much attention not only for environmental aspects [4,5,6,7], but also for interesting investigations such as biomedical applications [8], adhesives [9], optical properties [10], and structural analysis of the networks in the thermosets [11,12,13,14].

Especially, UV curable resins, a very important class of thermosets, are widely used as adhesives, printing plates, coatings, etc., due to their toughness and high reactivity. In general, multifunctional monomers having reactive units were irradiated to form UV cured resins in the presence of a photoinitiator.

The UV curing system is roughly categorized into three units, i.e., the radical system, cationic system, and anionic system. The radical system is mainly used for the conventional UV curing system. High mechanical strength, high solvent and heat resistance, short curing time, and energy savings were easily accomplished, therefore, the system is cost-effective. However, the demand for a higher reactivity and reduced oxygen inhibition still remain. Moreover, the analysis of polymer networks in photocured resins [11,12,13,14] is still challenging. In this study, we focused on the thiol-ene UV curing system which reduces oxygen inhibition, and a reworkable monomer, which was used for the analysis of the network structures.

**Figure 1:**
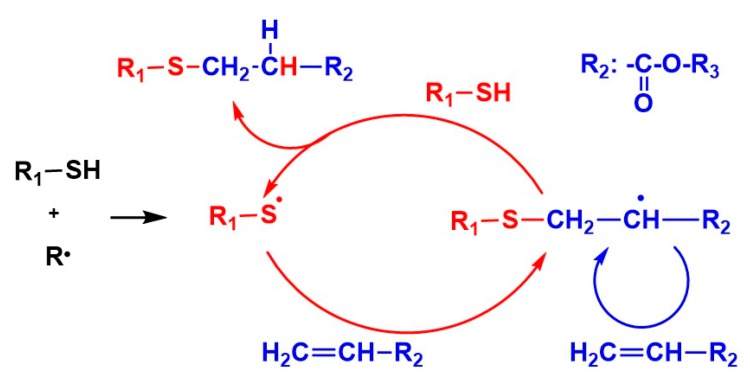
Reaction mechanism of thiol-acrylate system.

The thiol-ene system [14,15,16,17,18,19,20,21,22,23,24,25,26,27,28,29,30,31,32,33,34,35,36,37] has been extensively studied by Hoyle et al. [15,16,17,18,19,20] and Bowman et al. [21,22,23,24]. In the thiol-ene system, as shown in Figure 1, the sequential addition reaction of thiyl radicals to C=C double bonds followed by hydrogen abstraction from the thiol regenerates the thiyl radicals. The regeneration of thiyl radicals in the presence of oxygen gives tolerance to the oxygen inhibition. In the thiol-(meth)acrylate system (Figure 1), the conventional thiol-ene reaction and homopolymerization of (meth)acrylate simultaneously proceed [21]. The kinetics of the thiol-methacrylate system was investigated by Bowman et al. [21]. We focused on the investigation of the chain length in the thiol-(meth)acrylate system using a reworkable monomer. The investigation of the chain length of the methacryate photopolymerization using a reworkable monomer, a dimethacryate having anhydride linkages, was reported by Anseth [11]. This work motivated us to investigate the chain length of the thiol-(meth)acrylate system [14].

**Figure 2:**
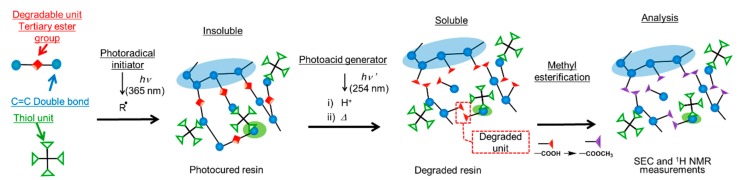
Schematic representation of analysis of network structures in this study.

In this paper, we extend the previous study [14] involving the analysis of polymer networks in the thiol-ene UV curing system using a reworkable monomer. A schematic representation of this study is shown in Figure 2. A reworkable monomer, which has both (meth)acryl units, and degradable units was mixed with multifunctional thiol compounds. UV curing of the blends of the reworkable monomer and the thiols was carried out by irradiation at 365 nm in the presence of a photoradical initiator. The tertiary ester linkages incorporated in the networks were cleaved by the photoinduced acid generated from the photoacid generator upon irradiation at 254 nm and followed by baking. The degraded compounds were (meth)acrylic acid derivatives and a diene which is vaporized and removed from the system. The analysis of the network structures was carried out by ^1^ H NMR and SEC measurements. The effects of formulations, curing conditions, and oxygen inhibition on the network structures of the cured resins were discussed.

## 2. Materials and Methods 

**Figure 3:**
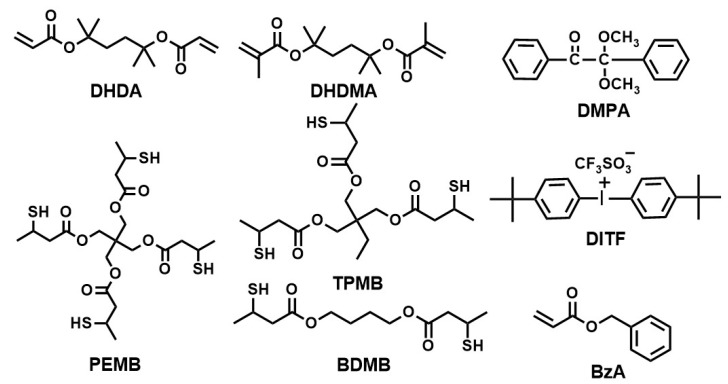
Chemical structures of compounds used in this study.

A photoradical initiator, 2,2-dimethoxy-2-phenylacetophenone (DMPA), a photoacid generator, di(*tert*-butylphenyl)iodonium trifluoromethanesulfonate (DITF), trimethylsilyldiazomethane (10 wt % solution in hexane), and a monofunctional monomer, benzyl acryate (BzA) were purchased from Tokyo Chemical Industries (Tokyo, Japan) and used as received. Reworkable monomers, 1,1′-(1,1,4,4-tetramethyl-1,4-butanediyl) diacrylate (DHDA) and 1,1′-(1,1,4,4-tetramethyl-1,4-butanediyl)dimethacrylate (DHDMA), were synthesized as already described [4]. A difunctional thiol, 1,4-butanediyl bis(3-mercaptobutyrate) (BDMB), a trifunctional thiol, trimethylol propane tris(3-mercaptobutyrate) (TAMB), and a tetrafunctional thiol, pentaerythritol tetrakis(3-mercaptobutyrate) (PEMB) were kindly donated by Showa Denko K.K. and used as received. The chemical structures of DHDA, DHDMA, BzA, BDMB, TAMB, PEMB, DMPA, and DITF are shown in Figure 3.

A mixture of monomers, a photoradical initiator, a photoacid generator, and thiol compounds (ca. 2 mg) was placed in a 5-mm-diameter aluminum pan. The thickness of the samples was about 200 μm. Irradiation at 365 nm was performed in air or under N_2_ using a Shimadzu UV-DSC system with a mercury-xenon lamp (Hamamatsu Photonics, LIGHTNINGCURE LC8, 200 W, Hamamatsu, Japan) in combination with a 365 nm bandpass filter. The cured sample was irradiated at 254 nm in air using an Asahi Spectra MAX-301 xenon lamp (300 W) (Asahi Spectra, Tokyo, Japan) in combination with a 254 nm bandpass filter. The intensity of the light was measured by an Orc Light Measure UV-M02 (ORC Manufacturing Co., Ltd., Machida, Japan). The sample pan was baked at 160 ℃ for 5 min on a conventional hot plate. The insoluble fraction was determined by gravimetry before and after dissolution of the sample in tetrahydrofuran (THF). The methylation of the samples was carried out by the reaction with trimethyldiazomethane for 2 h in toluene/hexane (1:1, *v*/*v*) at room temperature to give the methylated samples.

The transparency of the UV-cured samples was characterized by UV-vis spectroscopy taken by a Shimadzu UV-2400 PC (Shimadzu, Kyoto, Japan). More than 90% of the light was penetrated in all experimental conditions in this study. Photo-differential scanning calorimetry (photo-DSC) was carried out using a Shimadzu UV-DSC system (Shimadzu, Kyoto, Japan) to analyze reaction rate of the samples. The detailed description is appeared in Results and Discussion. Raman spectra were obtained by a JASCO RMP-315 (JASCO Corporation, Hachioji, Japan) to analyze the conversion of thiol and (meth)acryl functions in the samples as described in Results and Discussion. The SEC measurements were carried out to analyze the molecular weights of the degraded samples using an SEC system (JASCO PU-2080, JASCO Corporation, Hachioji, Japan) with polystyrene gel columns, TSKgel GMH_HR_-N, and TSKgel GMH_HR_-H. The molecular weights for the polymers were calibrated by poly(methyl methacrylate) standards. A preparative SEC was carried out to analyze the structures of the degraded samples using polystyrene gel columns, TSKgel G2000H_6_ and G1000H_HR_. A ^1^H NMR measurement was carried out using a JEOL ECX-400 (JEOL Ltd., Akishima, Japan) to analyze the chain lengths of the degraded samples. The detailed description is appeared in Results and Discussion.

## 3. Results and Discussion

### 3.1. Photocuring

**Figure 4:**
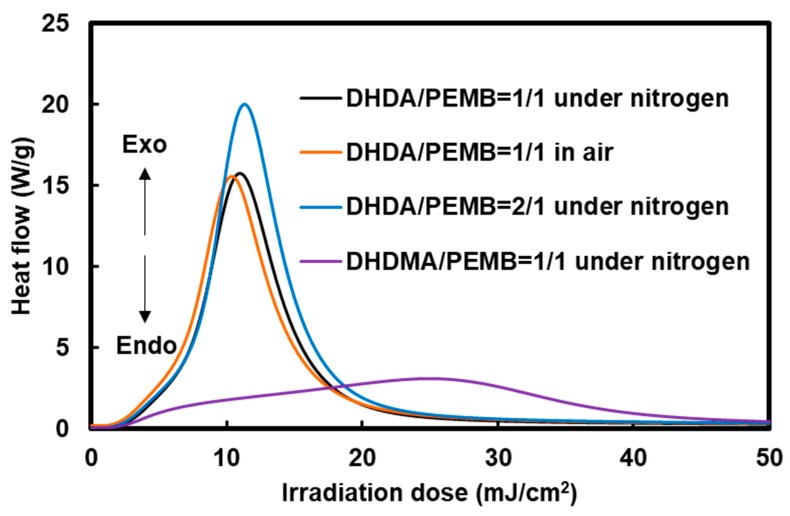
Photo-differential scanning calorimetry (photo-DSC) exotherms of the mixtures of 1,1′-(1,1,4,4-tetramethyl-1,4-butanediyl) diacrylate (DHDA) or 1,1′-(1,1,4,4-tetramethyl-1,4-butanediyl)dimethacrylate (DHDMA) and pentaerythritol tetrakis(3-mercaptobutyrate) (PEMB) containing 1 wt % 2,2-dimethoxy-2-phenylacetophenone (DMPA) and 1 wt % di(*tert*-butylphenyl)iodonium trifluoromethanesulfonate (DITF). Irradiation conditions: 0.50 mW/cm^2^ at 365 nm. The ratios in the figure show the functional ratio of the (meth)acryl and thiol groups.

The photocuring behavior of the mixture of (meth)acrylates, thiols, a photoradical initiator, and a photoacid generator was investigated using photo-DSC and Raman measurements. The photo-DSC measurements show the relative polymerization rates of the mixtures, which are useful to discuss the effect of the structure, formulation, and atmosphere on the photocuring reactivity. Figure 4 shows the Photo-DSC exotherms of the (meth)acrylate and thiol compounds mixtures containing 1 wt % DMPA and 1 wt % DITF.

As shown in Figure 4, the ratio of thiol and acryl groups was not strongly affected by the photopolymerization behavior. In terms of oxygen inhibition, a slight difference was observed when the DHDA/PEMB mixtures with a 1 to 1 functionality of the acryl/thiol units were used. Thus, there is little oxygen inhibition in the system. Photopolymerization using DHDA is faster than when using DHDMA, which is reasonable from the fact of the faster polymerization of common acrylates than methacrylates.

**Figure 5:**
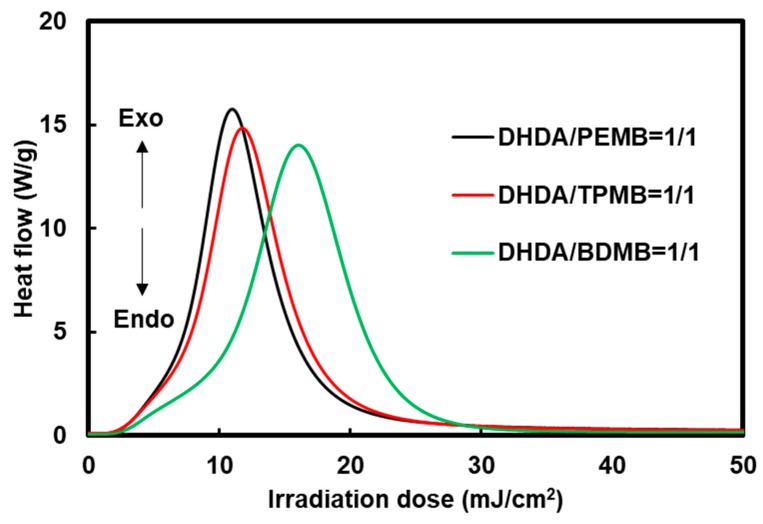
Effect of functionality of the thiol compounds on Photo-DSC exotherms of the mixtures of DHDA and thiol compounds containing 1 wt % DMPA and 1 wt % DITF. Irradiation conditions: 0.50 mW/cm^2^ at 365 nm in air. The ratios in the figure show the functional ratio of the acryl and thiol groups.

The effect of the functionality of the thiol compounds was also investigated by Photo-DSC exotherms as shown in Figure 5. The slightly low photopolymerization rate using BDMB compared to PEMB and TPMB is due to low viscosity of the cured sample which also has low crosslinking densities.

**Figure 6:**
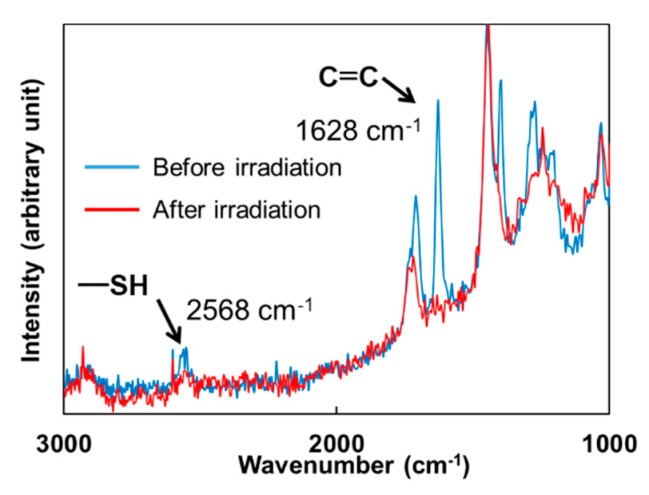
Raman spectra of the mixture of DHDA and PEMB (1/1, functional ratio of acryl and thiol groups) containing 1 wt % DMPA and 1 wt % DITF before and after irradiation. Irradiation conditions: 300 mJ/cm^2^ at 365 nm in air.

Conversion of the thiol-(meth)acrylate polymer networks was investigated by Raman spectroscopy. Figure 6 shows the Raman spectral changes of the DHDA/PEMB mixtures containing an equimolar content of the thiol and C=C double bond groups. The peaks ascribed to both the C=C double bond (1628 cm^−1^) and S–H bond (2568 cm^−1^) decreased during the irradiation. Conversions of the S–H groups and the C=C double bond groups were determined by the peak heights.

**Figure 7:**
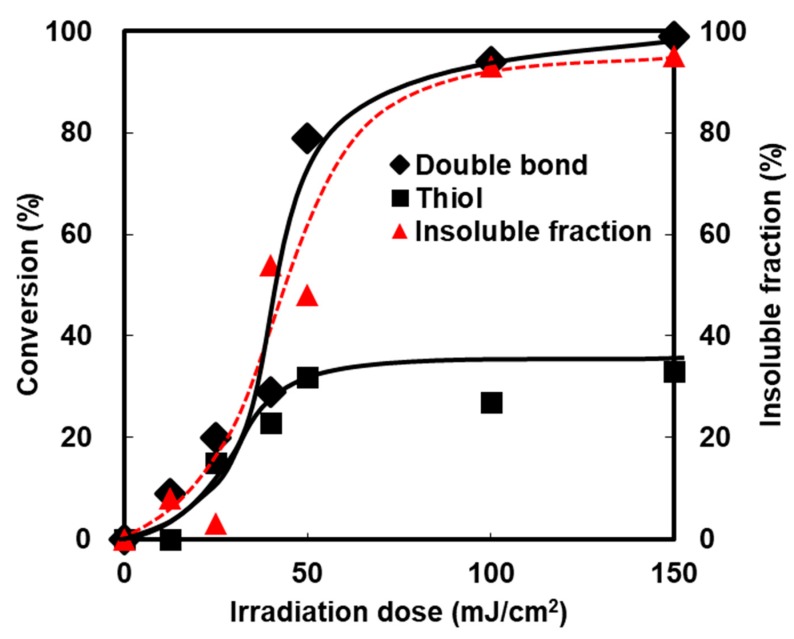
Conversion and insoluble fraction of acryl and thiol groups of DHDA/PEMB (1/1, functional ratio) containing DMPA (0.25 wt %) and DITF (1 wt %). Irradiation conditions: 0.50 mW/cm^2^ at 365 nm in air. The conversions were calculated by Raman spectroscopy.

The effect of the irradiation dose on the conversion of the methacryl and thiol groups was investigated and compared to the insoluble fraction of the samples (Figure 7). During an early stage of the photopolymerization, the thiol groups and the C=C groups were proportionally consumed. The homopolymerization of acrylates then exceeded the thiol-ene reaction when the conversions reached about 30%. The conversion curve suggested that the network structures in the DHDA/PEMB system mainly consist of two parts, i.e., thiol-ene structure and homopolymer of the acrylates. The insoluble fraction also increased with the irradiation dose. The insoluble fraction coincided with the conversion of the C=C groups.

### 3.2. Analysis of Chain Length

**Figure 8:**
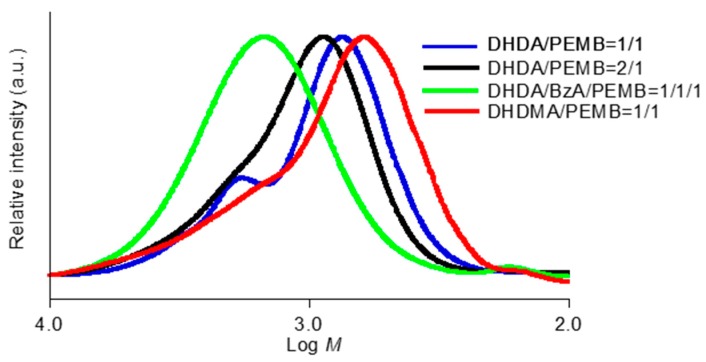
SEC profiles of degraded photocurable resins containing DMPA (1 wt %) and DITF (1 wt %). The ratios in the figure show the functional ratio of the acryl and thiol groups.

**Figure 9:**
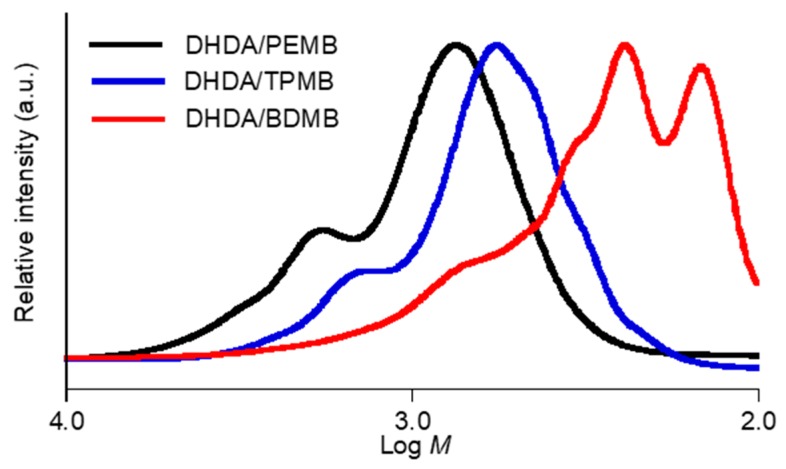
SEC profiles of degraded photocurable resins containing DMPA (1 wt %) and DITF (1 wt %). The functional ratio of the acryl and thiol groups was 1 to 1.

The analysis of the chain length was carried out using SEC measurements. The SEC profiles of the cured mixtures of (meth)acrylates and thiols containing DMPA and DITF after degradation are shown in Figure 8 and Figure 9. The weight-average molecular weight (*M*_w_), the number-average molecular weight (*M*_n_), and polydispersity (*M*_w_/*M*_n_) obtained from the mixtures of DHDA and PEMB which contain equimolar amounts of the C=C double bond and thiol groups, were 1200, 820, and 1.5, respectively. The obtained molecular weights do not correspond to the exact molecular weights because of the difference between the hydrodynamic volumes of poly(methyl methacrylate)s and those of the obtained compounds. The trial to investigate the relationship between the exact mass and the mass observed in SEC was unsuccessful using MALDI-TOF mass and GC mass spectroscopy. Thus, we discuss the values obtained in SEC qualitatively. The molecular weight (*M*_p_) calculated by the retention time which showed a maximum intensity in the SEC measurements of the sample from DHDA/PEMB (1/1, functional ratio) was 700. The small shoulder at around 1400 in the molecular weight is presumably due to dimer of main fraction as discussed below. The shift in the peaks was observed by changing the formulation of DHDA and PEMB. The *M*_p_, *M*_w_, *M*_n_, *M*_w_/*M*_n_ values obtained from the sample of DHDA/PEMB (2/1, functional ratio) were 920, 1400, 950, and 1.5, respectively. The increased values strongly support that the chain length in the UV cured resin was proportional to the ratio of the thiol and acryl groups. The *M*_w_/*M*_n_ value was not affected by the ratio of the thiol and acryl groups. Addition of reactive diluent BzA affected the SEC profile as shown in Figure 8. The *M*_p_, *M*_w_, *M*_n_, *M*_w_/*M*_n_ values obtained from the sample of DHDA/BzA/PEMB (1/1/1, functional ratio) were 1490, 1900, 1300, and 1.4, respectively. The enhanced reactivity and the increased chain length may be due to the low viscosity of the system. Using DHDMA instead of DHDA, slight low chain length was observed (620, 1200, 700, and 1.7 in *M*_p_, *M*_w_, *M*_n_, and *M*_w_/*M*_n_, respectively)_._

The effect of the functionality of thiol compounds was also investigated (Figure 9). The *M*_p_, *M*_w_, *M*_n_, *M*_w_/*M*_n_ values obtained from the sample of DHDA/TPMB (1/1, functional ratio) were 570, 770, 620, and 1.3, respectively. In addition, the *M*_p_, *M*_w_, *M*_n_, *M*_w_/*M*_n_ values obtained from the sample of DHDA/BDMB (1/1, functional ratio) were 250, 450, 260, and 1.7, respectively. We consider that the difference is mainly due to the effect of molecular weight (*MW*) of thiol compounds, PEMB (*MW* = 545), TPMB (*MW* = 441), and BDMB (*MW* = 294). The reaction mechanism shown in Figure 1 suggests that essentially one thiol compound is incorporated per one degraded compound. It is reasonable that, assuming that the chain length of acryl units in the degraded compound is same in the degraded samples, we can account for the difference of the molecular weights among these compounds. Thus, we consider that the functionality of the thiol compounds only slightly affects the chain length of acrylates.

**Figure 10:**
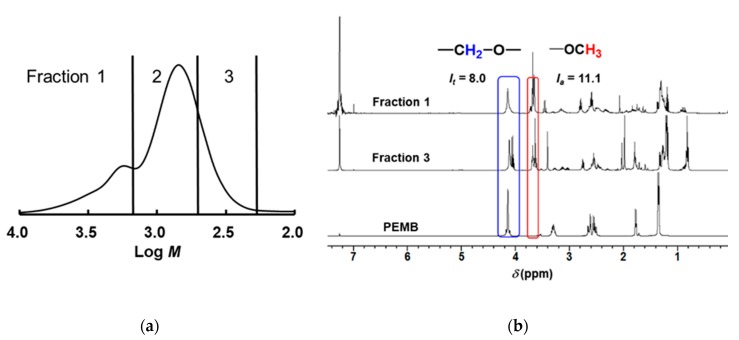
(**a**) SEC profiles of degraded DHDA/PEMB (1/1, functional ratio acryl and thiol groups) resins containing DMPA (1 wt %) and DITF (1 wt %). (**b**) ^1^H NMR spectra of each fraction of degraded DHDA/PEMB (1/1, functional ratio of acryl and thiol groups) resins containing DMPA (1 wt %) and DITF (1 wt %) after degradation in CDCl_3_.

Analysis of the degradation products in the cured thiol/(meth)acrylates was carried out. The degradation products were separated by their molecular weights using preparative SEC (Figure 10a). The fractionated compounds were analyzed by ^1^H NMR spectroscopy (Figure 10b). The information about the ratio of the thiol and acryl compounds is included in each fraction. In Fraction 1, the peak at a 4.2 ppm ascribed to methyleneoxy units in PEMB and the peak at 3.7 ppm ascribed to methoxy units in oligo(methyl acrylate) derived from DHDA were observed. The integrated peak area of each peaks are defined as *I*_t_ and *I*_a_, respectively. Using the values, we calculate the chain length, *ν*, the number of (meth)acryl units divided by the number of thiol units by the following equation:

*ν* = *I*_a_ / *I*_t_ × 8/3 (1)

In DHDA/TPMB and DHDA/BDMB system, the *ν* values were defined as the following equations:

*ν* = *I*_a_ / *I*_t_ × 6/3 (2)

*ν* = *I*_a_ / *I*_t_ × 4/3 (3)

In DHDA/BzA/PEMB system, the peak at 5.1 ppm ascribed to benzylic methylene units in BzA appeared in addition to the peak at 4.2 ppm ascribed to methyleneoxy units in PEMB and the peak at 3.7 ppm ascribed to methoxy units in oligo(methyl acrylate) derived from DHDA. When the integrated benzylic peak area was defined as *I*_b_, *ν* was defined as the following equation:

*ν* = (*I*_a_ / 3 + *I*_b_ /2)/(*I*_t_ / 8) (4)

The obtained *ν* value from Fraction 1 in Figure 10b was 3.7, and the value was identical to that obtained from Fraction 2. The observation indicates that Fraction 1 is consisted from the dimerization of the compounds that appeared in Fraction 2 by the termination of carbon to carbon, carbon to thiyl, and/or thiyl to thiyl radical coupling. The interpretation is good agreement with the SEC results as shown in Figure 8 and Figure 9. As for Fraction 3 in Figure 10b, the peak at a 4.2 ppm ascribed to methyleneoxy units in PEMB was not observed. Thus, we concluded that that the low-molecular-weight compounds are mainly the oligomers of methyl acrylate generated by a chain transfer reaction or from the terminal ends of the networks.

**Figure 11:**
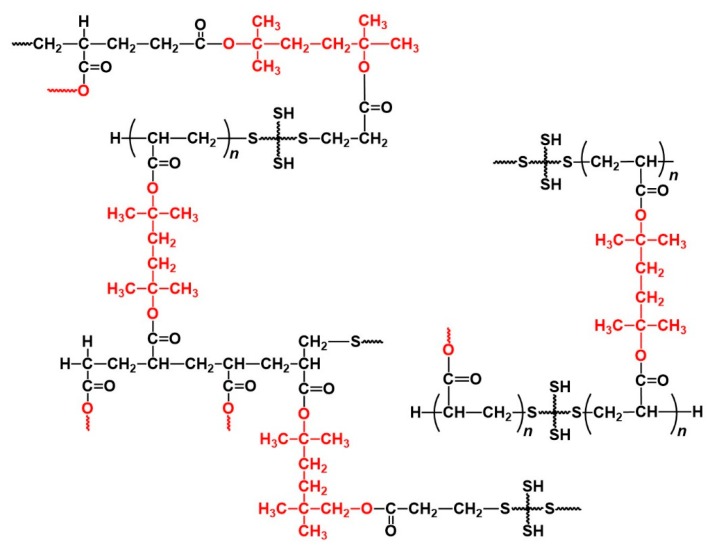
Plausible network structures of DHDA/PEMB (1/1, functional ratio of acryl and thiol groups) UV curing resin. The structures written in red were removed by decomposition.

Based on these results, we propose the reaction mechanism and formed structures of the polymer networks in the thiol-ene UV curing system using DHDA and PEMB. Upon irradiation at 365 nm, DMPA photolyzed to produce radicals which initiate the thiol-acrylate reaction of DHDA and PEMB. The formed networks consist of networks of the thiol-ene reaction and homopolymerization of the acrylates. The chain length of thiol-ene reaction is 1.7 to 14, and not very high due to the high concentration of the thiol compounds. The oligomers of the acrylates, whose molecular weight is lower than 300, also formed during the reaction. The networks were degraded by irradiation at 254 nm in the presence of the photoacid generator DITF and oligomers of the (meth)acrylic acid compounds, and the diene compounds are formed as shown in Figure 11. A summary of obtained results of the chain length in the thiol-(meth)acrylate UV curing system is shown in Table 1.

The degraded samples have molecular weights between 250 and 2700, and basically contain one thiol compound per one degraded molecule, which is consistent with the pathway of the thiol-ene reaction as shown in Figure 1. The molecular weights of the degraded resins increased with the functionality of the thiol compounds. The chain length dependence suggests that thiol compounds with a high functionality have a low reactivity due to steric hindrance. The chain lengths of the degraded networks were nearly proportional to the concentration of the (meth)acrylate monomers. The addition of BzA, a kind of reactive diluent, enhanced the reactivity and increased the chain length.

As for polydispersity values (*M*_w_/*M*_n_), relatively low values (1.3–1.7) were obtained in the experimental conditions in this study. It is very interesting that cured sample of DHDA/PEMB in air showed lower polydispersity value (1.3) compared to that under N_2_ (1.5). The low polydispersity value is due to suppressed dimerization observed in SEC measurements, which may be derived from the chain transfer reaction of carbon radical or thiyl radical to oxygen.

## 4. Conclusions

An analysis of the network structures of the thiol-ene UV curing resins was carried out using reworkable resins composed of di(meth)acrylate monomers having tertiary ester linkages. A mixture of (meth)acrylates, thiol compounds, a photoradical initiator, and a photoacid generator was cured by irradiation at 365 nm. The cured samples were degraded by heating after irradiation at 254 nm. SEC measurements and a ^1^H NMR analysis of the degraded samples were carried out after methylation by trimethylsilyldiazomethane. The crosslinking conditions strongly affected the network structures. The degraded samples have molecular weights between 250 and 2700. The molecular weights of the degraded resins increased with the functionality of the thiol compounds. Relatively low polydispersity values (*M*_w_/*M*_n_, 1.3–1.7) were obtained. The chain length dependence suggests that thiol compounds with a high functionality have a low reactivity due to steric hindrance. The chain lengths of the degraded networks ranged from 1.7 to 14, which was nearly proportional to the concentration of the (meth)acrylate monomers. The addition of reactive diluents enhanced the reactivity and increased the chain length.

## Figures and Tables

**Table 1:** Summary of chain lengths in thiol-(meth)acrylate UV curing system obtained in this study.

Formulation ^1^						[(meth)acryl Unit/Thiol Unit]	Conversion (%) ^2^		Insoluble Fraction (%)	*M*_p_^3^	*M*_w_^4^	*M*_n_^5^	*M*_w_/*M*_n_	*n* ^6^	Atmos-phere
DHDA	DHDMA	BzA	PEMB	TPMB	BDMB		(meth) acryl Unit	Thiol Unit							
1			1			1/1	99	35	99	700	1200	820	1.5	3.7	N_2_
1			1			1/1	96	33	99	740	1000	760	1.3	4.2	air
2			1			2/1	90	47	91	920	1400	950	1.5	9.8	N_2_
	1		1			1/1	92	29	100	620	1200	700	1.7	3.2	N_2_
1				1		1/1	98	40	98	570	770	600	1.3	2.7	N_2_
1					1	1/1	98	44	91	250	450	260	1.7	1.7	N_2_
1		1	1			2/1	96	25	96	1490	1900	1300	1.4	7.6	N_2_
1		3	1			4/1	97	14	95	2740	3200	2100	1.5	14	N_2_

^1^ The ratio of functional groups of (meth)acryl and thiol groups. All samples contained 1 wt % DITF and 1 wt % DMPA. ^2^ Determined by Raman spectroscopy. ^3^ Molecular weight calculated by the retention time which showed a maximum intensity in the SEC measurements calibrated by standard poly(methyl methacrylate)s. ^4^ Weight-average molecular weight. ^5^ Number-average molecular weight. ^6^ Chain length calculated by the number of (meth)acryl units divided by the number of thiol units determined by ^1^H NMR spectroscopy.
